# Facile hermetic TEM grid preparation for molecular imaging of hydrated biological samples at room temperature

**DOI:** 10.21203/rs.3.rs-2464569/v1

**Published:** 2023-02-14

**Authors:** Lingli Kong, Jianfang Liu, Meng Zhang, Zhuoyang Lu, Han Xue, Amy Ren, Jiankang Liu, Jinping Li, Wai Li Ling, Gang Ren

**Affiliations:** 1 The Molecular Foundry, Lawrence Berkeley National Laboratory, Berkeley, CA 94720; 2 School of Life Science and Technology, and Frontier Institute of Science and Technology, Xi’an Jiaotong University, Xi’an, China; 3 Department of Physics, University of California, Santa Barba, CA 93106; 4 Department of Biochemistry & Molecular Biology, Mayo Clinic, Jacksonville, FL 32224; 5 Université Grenoble Alpes, CEA, CNRS, IBS, F-38000 Grenoble, France

**Keywords:** liquid cell, environmental TEM, cell entry, virus-like particles, IPET

## Abstract

Although structures of vitrified supramolecular complexes have been determined at near-atomic resolution, elucidating *in situ* molecular structure in living cells remains a major challenge. Here, we apply a novel but simple liquid-cell technique, developed previously for real-time imaging of the dynamics at a liquid-gas interface, to image wet biological samples. With extra scattering from the liquid phase, the transmission electron micrographs show amplitude contrast comparable to that in negatively stained samples. Single-molecule domains are resolved in the protein complex GroEL imaged in buffer solution at room temperature. Moreover, various stages of virus cell entry, which are transient events with very few structural information to date, are also captured. Morphological details are reconstructed using the technique of individual particle electron tomography. These results demonstrate that this approach can be a valuable yet cost-effective technique complementary to other microscopy techniques for addressing important biological questions at the molecular level.

## Introduction

Since the development of the transmission electron microscope (TEM) in the 1930s, TEM imaging of biological samples in their aqueous environment has been a challenge^1,2^. Due to the strong interaction of electrons with matters, the electron microscope column is kept under vacuum to achieve a well-defined trajectory for the imaging electron beam. Any liquid phase sample will thus evaporate under the low vapor pressure, with consequences detrimental to the sample as well as the column vacuum.

Traditionally, aqueous biological samples are dried and chemically treated (negatively stained) for TEM studies^3,4^. Notably, the introduction of heavy metal stain changes the native environment of the protein sample and may also cause dissociation in the case of protein complexes. While negative staining can enhance image contrast and provide a useful first estimate of sample quality and shape, the technique does not reveal the true structure of biological samples.

To be able to study biological samples in their native hydrated state, cryo-electron microscopy (cryo-EM) has been developed^5–8^. Samples are frozen while inhibiting crystalline ice formation and the vitrified samples are imaged in sample stages cooled by liquid nitrogen. Using cryo-electron microscopes equipped with direct electron detectors, cryo-TEM has successfully resolved many three-dimensional structures of protein molecules and complexes at (near-)atomic resolution^9–11^. While such cryo-TEMs are available in many national or international user platforms, access is nevertheless limited.

To be able to image volatile samples with more widely available electron microscopes that operate at room temperature, the samples have to be isolated from the vacuum or hermetically encapsulated. For TEM imaging, the layer of sample has to be thin and the material for containing the liquid sample has to be transparent to electrons^1^. Collodion films^1^ have been used as a barrier between liquid samples and the vacuum in early studies^2^. Nonetheless, the low electron transparency of these films has led to a low-resolution rendering only with very limited structural details^12^.

Over the last decade, sophisticated TEM liquid cells using nano-fabricated microchips have been developed and have delivered remarkable results^13,14^. With these silicon-nitride microfluidic cells, material scientists have been able to achieve high-resolution real-time TEM imaging of nanocrystal interactions and chemical reactions in liquid environments^15–18^. Using scanning TEM (STEM), whole biological cells have also been imaged at nanometer resolution^19,20^. However, these complex microchips are expensive and must be mounted on specially designed holders^14^. The cost has prevented their regular application to biological samples, especially for preliminary studies, which often require extensive tuning of a large number of parameters in sample preparation procedures.

Another approach to seal liquid samples uses graphene monolayers deposited on TEM grids^21,22^. The liquid sample is trapped in a sandwich between graphene monolayers. This technique can achieve superior resolution as graphene monolayer is virtually transparent to the electron beam. Graphene also has excellent mechanical strength as well as thermal and electrical conductivity, which renders imaging more stable^23,24^. Nonetheless, graphene is inherently hydrophobic with a strong attraction between monolayers. Even though the hydrophobicity can be overcome by surface treatment, it is still difficult to control the amount of aqueous solution trapped in the random pockets formed between the monolayers.

In order to be able to perform molecular studies of liquid samples reliably with common room temperature TEM, we have recently developed a simple and reliable technique to hermetically encapsulate liquid samples. The dynamics of the vaporization of sodium, which was obtained through reduction reaction from sodium chloride, has been captured at nanometer spatial resolution using this technique^25^. Here, we adapt this technique to image biological samples. Using energy filtered TEM (EFTEM), electrons scattered in the liquid phase are eliminated, which gives rise to excellent contrast between solid proteins and their liquid environment. We explore the dose toleration of liquid samples by following the evolution of the sample with electron-beam irradiation. We show that nanometric structural features can be resolved in model protein complex GroEL in solution. We use HeLa cell infected by lentivirus as a model virus entry system. Using 3D reconstruction technique for individual particle electron tomography (IPET)^26^ , we identified virus particles fused with the HeLa cell membrane as well as particles inside the HeLa cell cytoplasm. By showing snapshots of viral-cell entry in this system, we demonstrate that our technique can be applied to study short-lived biological processes.

## Results

**Fig. 1:**
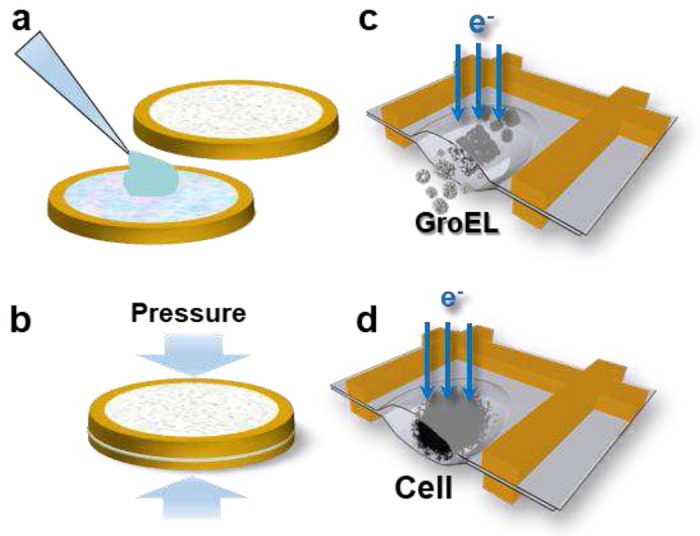
Schematic diagram of the assembly of the liquid cell. **a,** A small aliquot (~0.2 μL) of sample suspension is applied to the center of a Formvar-film-coated TEM grid and a second Formvar-film-coated TEM grid is carefully aligned to the first grid and placed on top of the first grid using an optical microscope. **b,** A controlled pressure is applied by a torque wrench to the sandwiched placed in a vise to squeeze out extra liquid and retain a desired liquid layer thickness. After the extra liquid is wicked away by a filter paper, a small amount of vacuum grease is applied on the circumference of the grid sandwich to seal the liquid cell. **c,** Liquid samples of protein GroEL trapped between the Formvar film in the grid squares can be imaged in the wet environment by the electron beam. **d**, Liquid samples of living cells trapped between the Formvar film in the grid squares can also be imaged in the wet environment by the electron beam.

We encapsulated biological samples using the liquid-cell technique we developed, which has captured the capillary waves in motion in molten Na at nanometer resolution^25^. Two standard 300-mesh TEM grids coated with Formvar film were used. Formvar coated TEM grids are available commercially but can also be prepared with straightforward procedures^25^. Fig. 1 summarizes the grid assembly process. Under an optical microscope, around 200 nanoliters of sample solution was applied to the Formvar film on the first TEM grid. A second Formvar coated grid was carefully aligned to the first grid under the optical microscope and placed with the Formvar side down onto the first grid with the sample. A well-controlled pressure was then applied to the sandwich by an accurizing torque wrench. After rounds of trial and error, we determined 0.55 bar to be the optimal pressure in our setup for achieving a thin liquid layer for TEM imaging without crushing the sample. The above steps were performed at a controlled relative humidity of > 90 % to prevent evaporation of the small volume of solution applied. Excess solution from the pressed grid sandwich was removed with a filter paper. The circumference of the sandwich was then sealed with vacuum grease and mounted onto a standard room temperature TEM sample holder to be introduced into the microscope. Imaging was performed on a Zeiss Libra 120 kV TEM equipped with an in-column energy filter.

Native GroEL sample in its buffer solution was loaded into the grid sandwich using the technique described above and imaged at room temperature. As a control, the same sample was also prepared by negative staining. The structure of the protein complex GroEL has been solved by X-ray crystallography as well as by single particle cryo-electron microscopy^8^. The chaperon protein consists of 14 monomers with D7 symmetry and has a molecular mass of ~800 kDa and dimensions of ~14 x 14 x 14 nm^3^, in which each monomer has a dimension of ~6 x 6 x 7 nm^3 8^.

**Fig. 2:**
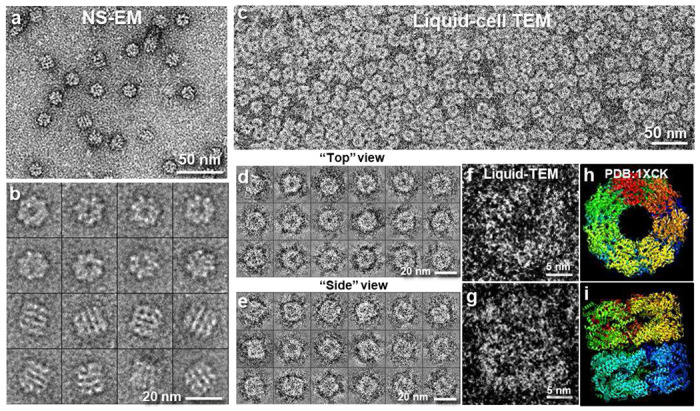
Liquid-phase TEM images of GroEL in TBS buffer compared to negatively stained (NS) images. **a**, GroEL sample stained with Uranyl Formate (UF). **b,** Representative particles in NS images. **c,** The same GroEL sample (identical batch as used in NS) encapsulated in our liquid cell and imaged at room temperature. The protein particles are distributed uniformly. Contrast is comparable to that in NS image. Contrary to NS, where only molecules attached to the film were retained, all GroEL molecules in the sample volume are present for imaging in the liquid cell. **d-e,** Representative GroEL particles in two orthogonal orientations**. f-g,** Magnified images of two particles in the different orientations **h-i,** the crystal structure of GroEL (PDB 1XCK) viewed along its *C_7_* symmetric axis and *C_2_* symmetric axis.

Fig. 2 compares the negatively stained GroEL sample (Fig. 2a–b) with the same GroEL sample imaged in buffer encapsulated using our technique (Fig. 2c–g). Fig. 2c shows evenly distributed GroEL particles true to its expected solution state. The liquid-cell sample yielded similar image contrast as the negatively stained sample, which suggests that the liquid phase scatters electrons more than the solid proteins. This effect was accentuated in our images because the scattered electrons were eliminated using zero-loss EFTEM in our experimental setup.

Nonetheless, unlike negative stain images (Fig. 2a and b), which show opaque surface features limited by the coating of heavy metal stain, the liquid-cell images are projections with information of the internal structure in the volume of the three-dimensional molecules. Top views and side views of the protein can be identified with structures (Fig. 2d and e) similar to those in cryo-images^8^. Seven domains can be picked out from magnified images of representative particles presented in their top view (Fig. 2f), which consistent with the structure determined by X-ray crystallography (Fig. 2h)^27^. Side views also show domain structure consistent with that determined by X-ray crystallography (Fig. 2g and i)^27^. Such structural details discerned in these wet sample are beyond those shown by other available structural techniques for wet samples to date.

The GroEL images in Fig. 2 were acquired with low-dose imaging at a dose of ~1.0 e^−^/Å^2^. In low-dose imaging, image acquisition areas between high-magnification and low-magnification settings are aligned by stage and image shift. Search for areas of interest is done at low magnification where the electron dose imparted on the sample is negligible. Focusing is done in an area distinct from the data collection area without exposing the latter. The area of interest is only exposed to significant and well calibrated dose (in this case ~1.0 e^−^/Å^2^) during the image acquisition at high-magnification. Low-dose imaging allows us to carefully follow the sample behavior as a function of electron beam irradiation.

**Fig. 3:**
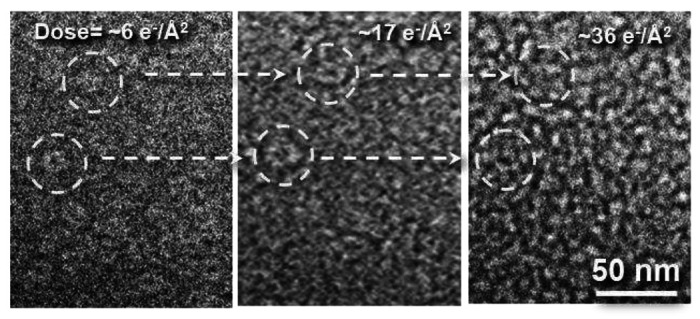
Effect of electron irradiation as a function of dose on GroEL in liquid cell.

Notably, when the cumulated dose increased to ~2.9 e^−^/Å^2^, degradation of the image quality could already be observed. Because of the stronger interaction with the electron beam, the wet sample suffer more severe radiation damage. When the cumulated dose reached 6 e^−^/A^2^ (Fig. 3), the GroEL molecules were barely distinguishable from the background. This dose is about 10× less than what is generally acceptable for vitrified protein samples in cryo-EM^28^. This shortcoming is expected for room temperature imaging as low temperature is known to slow down the processes induced by the electron beam^28^.

We next explore whether this new approach may be applicable to elucidate molecular mechanisms in living systems, such as virus entry. Virus entry is an important biological process with great significance in medicine. It is an attractive target for antiviral drugs as entry machinery is extracellular, which is easier for drug molecules to reach. Understanding virus entry is also essential to efficiently engineer virus that infect cells of choice (transduction retargeting) when virus is used as delivery vehicles for drug or genetic material. Despite the importance of virus entry, very little *in situ* structural information is available on the subject, presumably because of their transient nature^29,30^.

We used our technique to encapsulate a model of virus entry: HeLa cells mixed with a lentiviral protein transfer vector capable of infecting HeLa cells. Lentiviral vectors are virus-like particles derived from the human immunodeficiency virus type 1 (HIV-1) as recombinant vaccine vectors to deliver antigen-encoding gene sequences of pathogens into cells^31^. The lentiviral vector used in our experiments is pseudo-typed by the G-glycoprotein of vesicular stomatitis Indiana virus (VSV-G), which allows the infection of a broad range of cells, including HeLa cells^32^. This vector supports only one round of infection and does not replicate.

**Fig. 4:**
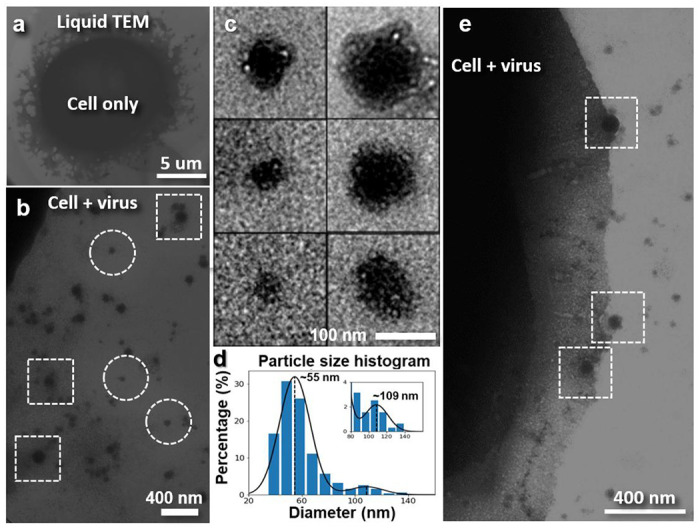
Liquid-phase TEM images of HeLa cells mixed with and without Lentiviral vectors in growth medium. **a,** A representative image **of a** HeLa cell only in growth medium. **b,** A representative image of the surrounding of a HeLa cell mixed with Lentiviral vectors. Part of the HeLa cell can be found on the upper left corner. Abundant small nanoparticles and larger particles (framed in circles and squares, respectively) are found. **c,** Representative images of small nanoparticles (left) and larger particles (right) show similar surface features. **d,** Histogram of the diameter of 315 particles shows a major peak at ~55 nm and a minor peak at ~109 nm. Size distribution of larger particles is consistent with that of Lentiviral vectors. **e.** The edge of a HeLa cell with virus-like particles (after high-pass filter). Three possible virus entry sites are boxed in green.

Fig. 4 shows images of the HeLa cells (Fig. 4a) and the Lentiviral vectors in the growth medium of the cell culture. Numerous particles with varied diameters but similar surface features are found in the solution background (Fig. 4b–c), which have not been observed in the control sample with HeLa cells alone. Statistical analysis showed two populations for these particles (Fig. 4d). Smaller particles have a peak diameter of ~55 nm, while larger particles have a peak diameter of ~109 nm.

The size distribution of the larger particles is similar to the size distribution of the lentiviral vectors (64 to 170 nm) observed by negative staining^33^ and cryo-EM^34^. Figure 4e shows a cell that appear to have these larger particles adhered to the cell surface, possibly in different stages of cell entry (Supplementary Video 2).

**Fig. 5:**
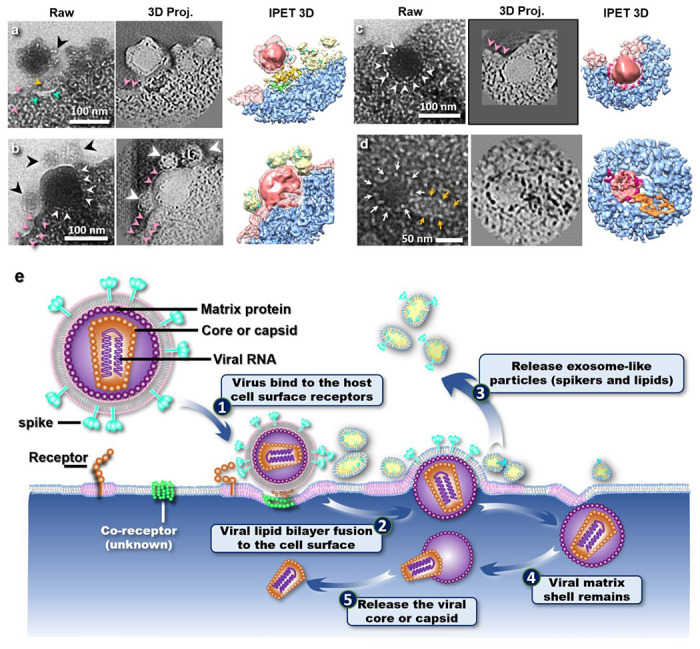
Liquid-phase TEM images (Raw), projection (3D Proj.) and 3D reconstruction of Lentiviral vectors interacting with HeLa cell membrane and hypothetical cell entry processes. **a,** Attachment (~7.2 nm resolution): Cell plasma membrane becomes concave in response to viral attachment. Orange and cyan arrows indicate two types of structures bridging the virus-like particle and the plasma membrane. The black arrow points to viral spikes that seem to be released from the viral surface. The pink arrows mark the abnormally thick membrane, which might represent a lipid raft. **b,** Penetration (~7.4 nm resolution): The particle is half embedded in the cell membrane. Note that the protruding spike proteins found on the particle in (**a**) are absent on the outer shell of this virus-like particle. On the other hand, three small nanoparticles (black arrows) of ~50 nm in diameter are found connected to the virus-like particle and appear to be coated with features similar in appearance to the spike proteins in (**a**). A tight coat of lighter density delimits the half virus from the HeLa cell (white arrows). A region of abnormally thick membrane is again observed (pink arrows). **c,** End of penetration (~6.5 nm resolution): No significant difference can be observed between the embedded particle and the particle during penetration; the particle remains round with a tight coat of lesser density. The pink arrows again indicate a region of abnormally thick cell membrane at the cell surface. On the other hand, no lipid membrane is found around the embedded virus. **d,** Uncoating (~6.4 nm resolution): endocytosed particle (white arrows) attached to a cone (orange arrows). All 3D volumes have been reconstructed by Individual Particle Electron Tomography (IPET). **e,** Schematic diagram of VSV-G mediated lentivirus vector entry into a cell inferred from (a-d). Membrane virus consists of a lipid bilayer of viral membrane decorated with surface spike protein trimers with a shell of matrix protein underneath, which surrounds a polygon or cone-shaped capsid with the viral RNA inside. To begin viral entry, the interaction between the viral spike proteins (cyan color) and the cell receptors on a lipid raft (pink color) triggers additional receptors to accumulate at the interface between the virus particle and the cell membranes to induce a concave curvature in the cell membrane. Upon opening a pore in the viral membrane, the virus particle penetrates the cell membrane by squeezing the viral membrane and unbounded spikes to form ~50-nm exosome-like protein-lipid nanoparticles under the association of lipid rafts. While the protein-lipid nanoparticles are released to the extracellular solution, the shell of matrix proteins remain intact throughout the membrane penetration process. The matrix protein shell is then gradually dissolved in the cytoplasm to release the capsid at a distance from the cell membrane.

To verify the 3D positioning of the particles relative to the cell membrane, we performed electron tomography on this sample region. As the size and appearance of these particles were consistent with those reported for lentiviral vectors by negative staining and cryo-EM studies, we supposed that these particles were lentiviral vectors. Fig. 5 shows magnified views of the particles from the zero-tilt projection of this sample region and the projections from the 3D reconstruction and its combination of density maps (that was superimposed its positive and negative density maps shown at ~7.2 nm resolution based on Fourier Shell Correlation of 0.5) after data processing with IPET. The 3D reconstruction showed that the virus-like particles were indeed in contact with the cell membrane, which was deformed to accommodate the curvature of the particles. These particles would be in the process of entering the cells as opposed to exiting because the Lentiviral vector is designed not to reproduce.

According to the relative contact area between the virus-like particles and the cell membrane, we attributed different stages of cell entry to the particles observed. The particle (~104-nm in diameter) in Fig. 5a appears to be connected to the cell surface through protrusions on the particle surface. The associated cell membrane forms a concave surface that envelopes around a quarter of the particle surface. Similar concave surface has been reported for VSV-G (GFP labeled pseudo-typed vectors) infecting T-cells observed by negative staining TEM ^35^. Alongside the protrusions (diameter of ~1-3 nm, black arrows indicated in Fig. 5a), other types of structures can also be observed bridging the large particle and the cell surface (cyan arrows and orange arrow in Fig. 5a).

Fig. 5b shows another spherical particle, with diameter ~130-nm consistent with the size of a lentivirus vector, half-embedded inside the cell membrane. The part of the particle outside of the cell membrane shows a smooth surface. The 7 nm small protrusions observed in Fig. 5a are absent on the surface of this particle. On the other hand, features reminiscent of the absent protrusions are found on three vesicle-like nanoparticles attached to the viral particle. These three nanoparticles have diameters of ~47 nm, ~64 nm, and ~65 nm, respectively (black arrow in Fig. 5b).

Fig. 5c shows another virus-like particle after just being embedded into the cytoplasm. The plasma membrane appears closed above the embedded particle. The particle had a slightly smaller diameter (~96 nm) than the ones observed in Fig. 5b. The spherical particle has a continuous coat of tiny particles of diameter ~5 nm (white arrows in Fig. 5c), which seems to be in direct contact with the cytoplasm.

We also found particles inside the cytoplasm that appeared to be internalized particles (Fig. 5d, Extended Data Fig. 2 and 3 and Supplementary Video 1), which were ~100 nm from the plasma surface. One of these particles is shown in Fig. 5d. This particle contains two distinct parts -- a globular portion (diameter of ~70 nm, indicated by the white arrows in Fig. 5d) adhered to a cone-shaped portion (of ~30 × ~45 nm, indicated by the orange arrows).

## Discussion

The results of this study demonstrate that the liquid cell we developed can be used as an efficient and simple technique for examining wet biological specimens at the molecular level. Resolution beyond 10 nm can be achieved using this technique, as shown in the GroEL images and the estimation with Fourier shell correlation in the tomograms. Whole-cell samples can be examined in the culture medium at room temperature without introducing staining, air-drying or plastic embedding artifacts. Importantly, the assembled liquid cells can be mounted on any standard TEM holder for large-scale screening.

Several precautions should be taken when performing liquid-cell TEM. Deficient sealing can cause sample leak, which will degrade the column vacuum. Glue types and sealing procedures are important factors in determining the seal quality. For optimal image quality, other key factors to consider include film materials, window size (grid mesh size), liquid thickness, and illumination dose. Our results also confirm that liquid samples are extremely sensitive to radiation damage. A high-speed, high-sensitivity detector will thus be appropriate for high-resolution liquid-cell imaging.

We showed that liquid-cell TEM on the biological samples gives comparable contrast as negatively stained samples, especially when coupled with EFTEM. Compared to amorphous ice, water molecules in liquid at room temperature possesses a lot more degrees of freedom. The additionally available vibrational modes in liquid compared to phonons in solids allow a broader spectrum of energy exchange with incoming electrons. Moreover, traversing electrons are more likely to encounter an atom in liquid with constantly moving molecules than in solid with stationary molecules. Liquid water thus scatters electrons more than solid sample made up of elements of similar atomic weight ^36,37^. On the other hand, evaporation might have occurred despite of our precautions, which is not surprising considering the small volume applied. In such a case, salt concentration in the growth medium would have increased and might have also contributed to the increased scattering. This effect becomes more prominent with EFTEM as scattered electrons were eliminated.

The results on the HeLa cells with Lentiviral vectors showed possible stages of viral cell entry (Fig.5a–d) and led us to propose a highly speculative infection pathway of VSV-G initiated virus entry (Fig. 5e). We speculate that the 7 nm protrusions (black arrows indicated in Fig. 5a) to be the viral surface spike proteins (glycoprotein VSV-G). Glycoprotein VSV-G are known to interact with cell receptors in cell entry, as in the case of VSV-G binding to low-density lipoprotein receptors (LDL-R)^38^. The additional observed structures (cyan arrows and orange arrow in Fig. 5a) could therefore be cell receptors and co-receptors^39^ that trigger the initiation of the membrane fusion process.

**Extended Data Fig. 1:**
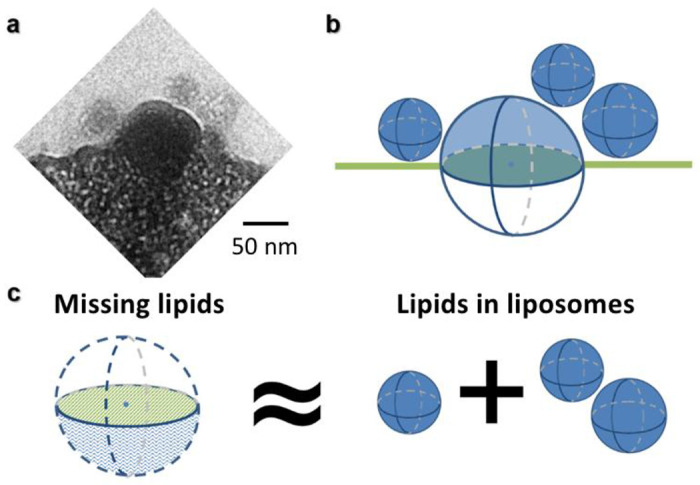
Calculation of excess lipid during fusion of membrane virus and cell **a**, The viral particle is half embedded in the cell membrane (Fig. 5b in main text). **b,** Schematic representation of the viral particle and the surrounding nanoparticles. **c,** The area of fused membrane (missing lipids), 27,980 nm^2^ is the sum of the area of the lower hemisphere of the viral particle (diameter D = 109 nm, corresponding to the peak of the larger particle population size in Fig. 4d), *i.e.* ~18,653 nm^2^ (πD^2^/2) plus the cell membrane area that was occupied by the virus, *i.e.* ~9,327 nm^2^ (πD^2^/4). Interestingly, the fused membrane area equals to ~84% of sum of the surface area of the three attached nanoparticles (diameters ~47 nm, ~64 nm and ~65 nm, respectively, close to the peak of ~58.6 nm for the smaller particle population size), which is ~33,064 nm^2^. The near-perfect matching to the surface area supports the hypothesis that the fused membrane has been released in the form of liposome nanoparticles (Fig. 5b).

We suggest that the nanoparticles observed in Fig. 5b are liposomes formed by the virus membrane with the spike proteins when the virus-like particle destabilizes the cell plasma membrane during insertion. Lipid rafts involved in virus entry *via* localization of cell receptor for viral entry^40^ might have reduced the local fluidity of the cell membrane. In fact, if the virus membrane was incorporated into the plasma membrane, wrinkles or other features conforming with the increase in the local membrane surface area should be observed. We had not observed any such features in our experiments. Indeed, simple estimation showed that the fused viral/cell membrane had a similar surface area to the total surface area of the three nanoparticles (Extended Data Fig. 1). We thus propose that liposomes are formed by the excess lipids from the viral membrane upon virus entry. This scenario would also explain the large population of small particles in the solution (Fig.4b), which were not observed in the solution of the HeLa cell sample without the lentivirus (Fig. 4a).

We also observed that the virus-like particles stayed spherical in shape after being embedded in the cell, suggesting that the release of the nucleocapsid happens inside the cell and not at the fusion site with the plasma membrane. The spherical particle has a continuous coat of tiny particles of diameter ~5 nm (white arrows in Fig. 5c). We interpret this coat to be the layer of matrix protein p17 exposed after the virus surface membrane envelope was released outside the cell as liposomes with the spike proteins. The matrix protein p17 encapsulates the cone-shaped nucleocapsid composed of the protein p24^41^, which encapsulates viral RNA condensed with ribonucleoproteins and enzymes^42^. The observation that the embedded virus remained spherical in shape and not cone-shaped implied that the viral capsid with matrix protein p17 subsisted the process of membrane fusion.

The particle observed in Fig. 5d further suggests that the release of the core happens inside the cytoplasm away from the plasma membrane. This particle contains two distinct parts -- a globular portion (diameter of ~70 nm, indicated by the white arrows in Fig. 5d) adhered to a cone-shaped portion (of ~30 × ~45 nm, indicated by the orange arrows). The two portions might correspond to the p17 matrix and the p24 nucleocapsid, respectively.^7,43^

The proposed entry pathway differs from current conceptions of virus entry, which involve endosome or the release of the capsid at the plasma membrane entry site. If the nanoparticles observed here at the virus entry site are indeed liposomes formed by the extra lipids upon fusion of the plasma membrane and the viral membrane (**see** Extended Data Fig.1), the matrix protein would be in direct contact with the cytoplasm upon entry as shown in Fig. 5c. Fig.5c–d further suggest that the capsid stays inside the intact matrix shell after entry and is released into the cytoplasm in the interior of the cell. Intermediate stages in this scenario would highly resemble those in virus production and egression. Higher resolution images (e.g. cryo-TEM with direct electron detectors) or immunolabelling will be necessary for future studies. The presence of liposomes with spike proteins, which might have implications in the host immune response, should also be further investigated.

## Methods

### Biological sample preparation

The Lentivirus was the third-generation virus that was packaged with three co-transfection vectors, plasmids pLKO.1, psPAX2, and pMD2.G, in which pMD2.G contained genes for envelope proteins—the G glycoprotein of vesicular stomatitis Indiana virus (VSV-G). The VSV-G protein allows the infection of a broad range of cells, including HeLa cell^44^. HeLa cells were grown in Gibco minimum essential media (MEM, containing 10% FBS) at 37 °C with 5% CO_2_, and virus-infected cells were prepared by incubating HeLa cells with virus at a ratio of 1:50 (1 cell to 50 virus) for 12 hours. The control GroEL used for negative staining imaging was provided by Dr. Scott Stagg’s laboratory: the protein at a concentration of ~0.5 mg/mL in Tris-buffered saline (TBS, 50 mM Tris, pH 7.4, 50 mM KCl, and 1 mM DTT) was diluted and stained by following the reported optimized negative-staining (OpNS) protocol^4^ and then imaged by a Zeiss Libra 120 TEM (Carl Zeiss NTS) using a Gatan UltraScan 4k x 4k CCD.

### Liquid-phase TEM specimen preparation

The method to sandwich the biological samples follows the published protocol ^25^. Briefly, ~0.2 μl of sample solution (native liquid state, label-free, without any staining) was deposited and then sandwiched between two layers of 300-mesh TEM Formvar-coated copper grids (~3 mm in diameter, ~100 μm in window diameter) at room temperature with a humidity level of >90%. The sandwiched grids were compressed together under a pressure of ~8 psi (~0.55 bar) for ~20 seconds. Any excess solution was then removed by filter paper before the edge of the sandwiched grids was sealed with high vacuum grease (DuPont Molykote, USA Lab) and then mounted on a regular TEM holder.

### Optimizing TEM imaging

The liquid-phase TEM grids containing label-free biological samples were examined at room temperature using a Zeiss Libra 120 Plus TEM (Carl Zeiss NTS) operated at a high tension of 120 kV with 20 eV in-column energy filtering. Micrographs were acquired on a Gatan UltraScan 4k × 4k CCD using a defocus up to 12 μm and magnification from 1,000× to 80,000× (each pixel of the micrographs corresponded to 107 Å to 1.48 Å, respectively, in the specimen). The radiation damage on the GroEL sample was tested using an illumination dose in the range from ~1 to ~36 e^−^/Å^2^ under magnification of 40,000× −80,000× and defocus up to −3 μm. Approximately 300 micrographs were acquired under low-dose conditions, a magnification of 80,000x, and defocus of ~0.1 – ~1.0 μm. Approximately 100 micrographs of HeLa cells were acquired under low-dose conditions at a magnification of 1,000× – 20,000×.

### Electron tomography (ET) tilt series acquisition

The liquid-phase EM holder for imaging HeLa cells was tilted at angles ranging from −36° to +60° in steps of 1° controlled by both Gatan tomography software and fully mechanically controlled automated electron tomography software^45^, which were preinstalled in the microscope (Zeiss Libra 120 TEM). The TEM was operated at 120 kV with a 20 e^−^V energy filter. The tilt series were acquired by a Gatan Ultrascan 4k × 4k CCD camera with a defocus of ~11 ~m and a magnification of 1,000× and 10,000× (corresponding to 107 Å and 10.7 Å per pixel, respectively, in the specimen). The total illumination electron dose for a tilt series was ~30 e^−^/Å^2^.

Due to the heavy overlapping of cell surface membranes, it was difficult to clearly identify the virus near the cell surface boundary. We therefore searched and found a rectangular shaped cell (~17 μm × ~6 μm in dimension, Extended Data Fig. 4 **and Supplementary Video 2** that contained a relatively smooth surface membrane. Two chain-shaped densities were present near the center (Extended Data Fig. 4 **and Supplementary Video 2**). These densities remained constant in shape and size during the tilt series acquisition at low magnification (~1,000 ×), suggesting that these objects were unlikely to be artifacts from radiation damage but represented real features inside the cell. The IPET 3D reconstruction confirmed two chain-shaped densities with similar size and shape near the center of this rectangular shaped cell (Extended Data Fig. 4c and **Supplementary Video 1** and **2**). The overall morphology was similar to that observed by light microscope for a dividing cell with two copies of chromosomes ^46^.

### Image preprocessing

The defocus and astigmatism of each micrograph in the tilt series were measured and the contrast transfer function (CTF) of each image was corrected using EMAN *CTfit* software^47^. Prior to CTF correction, X-ray speckles were removed, and micrographs with distinguishable drift were excluded. To remove the strong background and enhance high-resolution details, each micrograph was filtered using a Gaussian boundary high-pass filter (at a resolution range of 200 nm to 500 nm). Micrographs in the tilt series were initially aligned with the IMOD software package^48^. The CTF was then corrected by TOMOCTF^49^. The tilt series of virus-like particles in square windows of 128 to 360 pixels (~137 to ~386 nm) were semi automatically tracked and windowed by IPET software^26^.

### Statistical analysis of virus-like particle sizes

For statistical analysis of particle sizes, all particles surrounding the cell (~315 in total) were selected and windowed. Each particle’s area was measured by Python Open CV. The histograms of the particles with diameters above and below 90 nm were fitted by Gaussian curves. The curves were merged based on the weights according to their particle numbers using a Python code, named curve_fit from the python module scipy.optimize to fit our data.

### IPET 3D reconstruction

The targeted particles (HeLa cells, viruses, and nanoparticles) in the tilt series were reconstructed by the IPET software^26^. In brief, a circular mask with a Gaussian edge was applied to each image, followed by 3D reconstruction via an iteration refinement process with a series of soft-boundary masks and filters. To display the objects with positive or negative contrast, a superimposed 3D density map was generated by combining the positive contour map of the final IPET 3D density maps with its negative contour map by using the Chimera software^50^. The resolution of the final 3D map was estimated based on the intra-Fourier shell correlation (FSC)^26^. Briefly, the aligned images were then split into two groups (odd- or even-numbered tilting index) to generate two 3D reconstructions. An FSC curve is computed using the two reconstructions and the frequency at which the FSC curve fell to a value of 0.5 was used to represent the resolution.

## Extended Data

**Extended Data Fig. 2:**
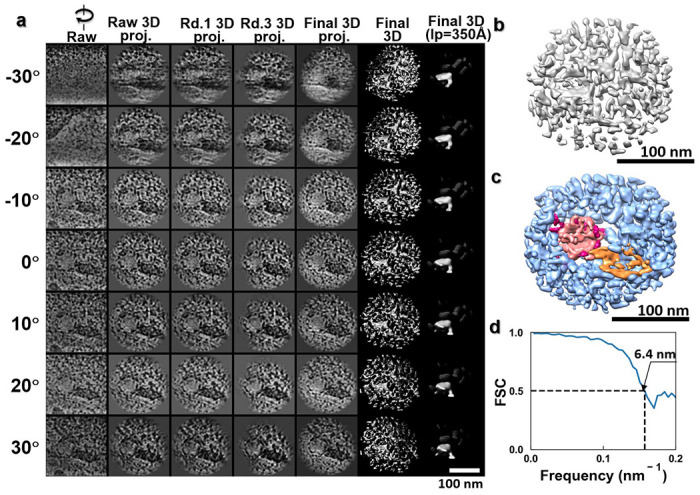
IPET 3D reconstruction of a broken-virus particle in the cytoplasm. **a**, Seven tilted views (first column), their corresponding projections on the intermediate 3D reconstructions from major iterations (second to forth columns), and the final 3D density map (fifth column) are shown. **b**, The final 3D density map. **c**, The superimposed 3D map that combines the positive map, which highlights the virus-like particle structure and the negative map, which highlights the internal capsid-like particle structure. **d**, The FSC curve shows that the resolution of the final 3D reconstruction is ~6.4 nm.

**Extended Data Fig. 3:**
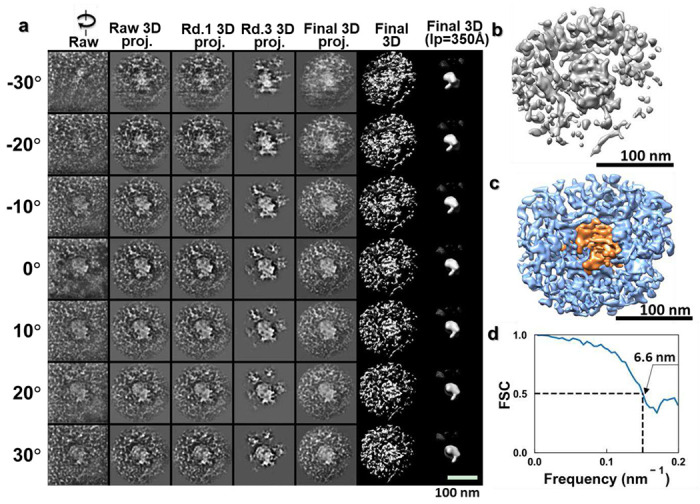
IPET 3D reconstruction of a broken-virus particle in the cytoplasm. **a**, Seven tilted views (first column), their corresponding projections on the intermediate 3D reconstructions from major iterations (second to forth columns), and the final 3D density map (fifth column) are shown. **b**, The final 3D density map. **c**, The superimposed 3D map that combines the masked map for showing the capsid-like particle boundary structure and the original map for showing the surrounding densities. **d**, The FSC curve shows that the resolution of the final 3D reconstruction is ~6.6 nm.

**Extended Data Fig. 4:**
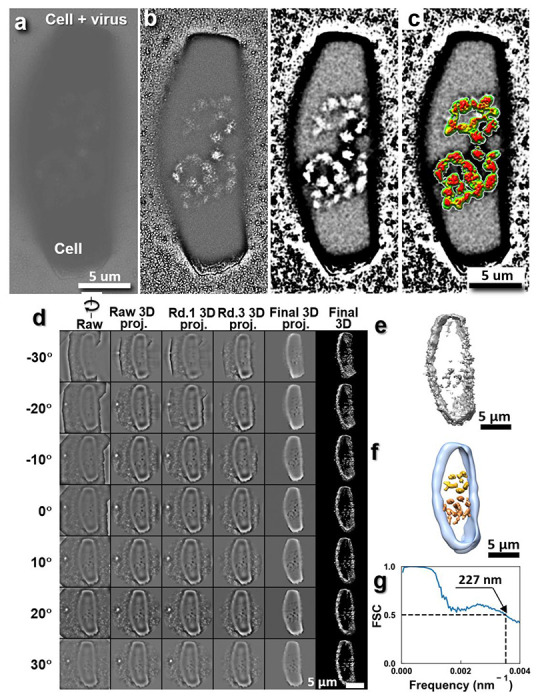
Liquid-phase TEM images and 3D reconstruction of a HeLa cell in liquid growth medium. **a,** Raw images of a rectangle shaped HeLa cell, **b,** Detailed internal cell structure shown by high-pass filtered (left panel) and contrast enhancement image processing (right panel). **c,** Detailed internal chromosome-like structure shown by superimposing with its tomography image of the center density portion. **d**, Seven tilted views (first column), their corresponding projections on the intermediate 3D reconstructions from major iterations (second to forth columns), and the final 3D density map (fifth column) are shown. **e**, The final 3D density map. **f**, The superimposed 3D map that combines the positive map, which highlights the cell boundary structure and the negative map, which highlights the internal chromosome structure. **g**, The FSC curve shows that the resolution of the final 3D reconstruction is ~227 nm.

## Data Availability

The liquid TEM density maps of three viruses are available from the EM databank as EMD-25890, 25894 and 25895.
